# A statistical analysis of vaccine-adverse event data

**DOI:** 10.1186/s12911-019-0818-8

**Published:** 2019-05-28

**Authors:** Jian-Jian Ren, Tingni Sun, Yongqun He, Yuji Zhang

**Affiliations:** 1 Statistics Program, Department of Mathematics, University of Maryland, College Park, 20742 MD USA; 2 Department of Microbiology and Immunology, University of Michigan Medical School, Ann Arbor, 48109 MI USA; 3 Department of Epidemiology and Public Health, University of Maryland School of Medicine, Baltimore, 21201 MD USA

**Keywords:** Bacteria vaccine, Correlation coefficient matrix, Data visualization, Inactivated vaccine, Live vaccine, Neighboring method, Virus vaccine

## Abstract

**Background:**

Vaccination has been one of the most successful public health interventions to date, and the U.S. FDA/CDC *Vaccine Adverse Event Reporting System* (VAERS) currently contains more than 500,000 reports for post-vaccination adverse events that occur after the administration of vaccines licensed in the United States. The VAERS dataset is huge, contains very large dimension nominal variables, and is complex due to multiple listing of vaccines and adverse symptoms in a single report. So far there has not been any statistical analysis conducted in attempting to identify the cross-board patterns on how all reported adverse symptoms are related to the vaccines.

**Methods:**

For studies of the relationship between vaccines and reported adverse events, we consider a partial VAERS dataset which includes all reports filed over a period of 24 years between 1990-2013. We propose a *neighboring method* to process this dataset for dealing with the complications caused by multiple listing of vaccines and adverse symptoms in a single report. Then, the combined approaches based on our neighboring method and novel utilization of data visualization techniques are employed to analyze the large dimension dataset for characterization of the cross-board patterns of the relations between all reported vaccines and events.

**Results:**

The results of our analysis indicate that those events or symptoms with overall high occurrence frequencies are positively correlated, and those most frequently occurred *adverse* symptoms are mostly uncorrelated or negatively correlated under different bacteria vaccines, but they are in many cases positively correlated under different virus vaccines, especially under flu vaccines. No particular patterns are shown under live vs. inactive vaccines.

**Conclusions:**

This article identifies certain cross-board patterns of the relationship between the vaccines and the reported adverse events or symptoms. This helps for better understanding the VAERS data, and provides a useful starting point for the development of statistical models and procedures to further analyze the VAERS data.

## Background

Vaccination has been one of the most successful public health interventions to date. However, the use of vaccine sometimes comes with possible adverse events. Since 1990, the U.S. FDA/CDC *Vaccine Adverse Event Reporting System* (https://vaers.hhs.gov/data/datasets.html) (VAERS) has received 530,716 case reports by the end of 2016 for post-vaccination events that occur after the administration of vaccines licensed in the United States. The primary objectives of VAERS are to detect new, unusual or rare vaccine adverse events or symptoms; monitor increase in known adverse events; identify potential patient risk factors for particular types of adverse events; assess the safety of newly licensed vaccines; etc.

Each VAERS report includes the following information of an individual: patient ID, place of vaccination, age, gender, vaccines administrated, adverse events or symptoms observed, time between vaccination and adverse event onset, etc. The VAERS data at FDA site are not ready for statistical analysis without being processed, because each report lists adverse events or symptoms in the form of non-regulated words or phrases, and often contains multiple listing of symptoms along with multiple listing of vaccines. Taking into account the possible multiple listing of vaccines and adverse events or symptoms in one report, a well processed dataset file based on current 530,716 case reports during 1990-2016 is estimated to have 2,000,000 - 3,000,000 rows. Thus, this is a big and complicated data set.

**Challenges:** In addition to the large data size issue, as the key components for our research interests the vaccine variable *V* and symptom variable *Z* in VAERS data are nominal variables, and the already very large dimension of symptom variable *Z* (i.e., the total number of different categories) can still increase as more reports are being filed each year. In statistical literature, we have few tools for such kind of data analysis involving nominal categorical variable with unlimited dimension. Another big complication of the VAERS data is due to above mentioned multiple listing of vaccines administrated and multiple listing of adverse symptoms in one single VAERS report. For instance, one report may list vaccines A and B and list adverse symptoms C, D and E. In such a case, we do not exactly know which symptom was triggered by which vaccine. Unfortunately, such huge complication in VAERS data will continue until one vaccination per time is enforced in U.S. Thus, this posts great challenges for the analysis of vaccine data.

Dr. He of this project team was the primary developer of the vaccine ontology. Recently, he and Dr. Zhang (co-author of this article) along with other collaborators have conducted some network-based studies on the VAERS data to summarize and analyze the vaccine-adverse event association [1–3], and have done some ontology-based comparative analyses on the adverse event associated with killed and live influenza vaccines [4]. But these works are not the statistical analysis in the usual sense.

It is well-known that before a particular vaccine was marketed, clinical trials had already identified some adverse symptoms or events associated with such vaccine. However, this is not equivalent to the cross-board patterns of the relations between vaccines and adverse events or symptoms. With huge VAERS data accumulated at this point, the analysis of such cross-board patterns becomes possible, but so far there has not been any statistical analysis conducted in attempting to identify the cross-board patterns on how all reported adverse symptoms are related to the vaccines. Characterizing such cross-board patterns is of importance on its own for better understanding the VAERS data, and would provide insights for developing statistical models and procedures for further analysis of VAERS data. In particular, the characterization of cross-board patterns is in fact a method of using all available data together to deal with the big complication problem in VAERS data caused by aforementioned multiple listing of vaccines and adverse symptoms in a single report; that is one single report with multiple listing makes it impossible for us to know exactly which symptom was triggered by which vaccine, but putting all reports with related information together can lead us to identify cross-board patterns on the relationship between vaccines and adverse symptoms.

In this article, a partial VAERS dataset is considered for characterizing the cross-board patterns of the relationship between all reported vaccines and all reported adverse symptoms or events. We propose a *neighboring method* to process the raw VAERS data, and we analyze this processed large dimension dataset via novel utilization of data visualization techniques [5] developed for the big data analysis.

## Methods

### Data

#### Data processing

As mentioned above, the original VAERS data at FDA site are not ready for statistical analysis without being processed. Here, for the study of causal relationship between all reported vaccines and all reported events or symptoms, we consider a partial dataset of VAERS data which was based on all 407,453 reports filed over a period of 24 years between 1990–2013. This partial dataset is processed using our proposed *neighboring method* into the following form of *n*=277,698 vectors:

1 $$ \boldsymbol{\mathbb{V}}=(\mathbb{Y}, V, Z, W),  $$

where $\mathbb {Y}$ represents year; *V* represents the vaccines with a total of 72 different types; *Z* represents the symptoms, such as abdominal pain, anxiety, autism, blindness, coma, depression, eye disorder, fatigue, headache, inflammation, swelling, vomiting, etc., with a total of 7368 different symptoms; and *W* represents the total number of occurrences of symptom *Z* after vaccine *V* was administrated during year $\mathbb {Y}$. For instance, vector (1991,DTP, Pyrexia,2107) means that during year 1991, the occurrence of symptom *Z* =[Pyrexia] after vaccine *V* =[DTP]’s being administrated was listed in a total of *W* =2107 reports; vector (2003, DTAP, Injection Site Erythema, 1797) means that during year 2003, the occurrence of symptom *Z* =[Injection Site Erythema] after vaccine *V* =[DTAP]’s being administrated was listed in *W* =1797 reports; and vector (2009,FLU(H1N1), Rash,547) means that during year 2009, the occurrence of symptom *Z* =[Rash] after vaccine *V* =[FLU(H1N1)]’s being administrated was listed in *W* =547 reports. In this paper, our processed dataset only includes those vectors with positive frequency variable *W*.

#### Neighboring Method

For the case of a report with multiple listing of vaccines and events or symptoms as aforementioned, it is processed as follows. If a report lists vaccines A and B and lists symptoms or events C, D and E, each of symptoms C, D and E is counted once for each of vaccines A and B, respectively, for frequency variable *W* in Eq. (1). The description and rationale of our proposed neighboring method are:

(i) From this one single report, we do not know whether symptom C was triggered by vaccine A or vaccine B or both; the same goes with symptoms D and E;
(ii) Because of (i), we count the occurrence of symptom C under vaccine A once, adding 1 into the corresponding frequency variable *W* in Eq. (1); also count the occurrence of symptom C under vaccine B once; and do the same for symptoms D and E for the same reasons;
(iii) The resulting processed data in the form of Eq. (1) as a whole allow us to use all reports including, say, symptom C and vaccine A, to study the cross-board patterns of the relationship between all reported vaccines and all reported adverse symptoms, which contain symptom C and vaccine A as a pair. This is the idea of using all neighboring information to study the relation of a particular pair.

#### Additional Notes

Some of the VAERS reports considered in our studies here contain errors or incomplete information. For instance, some reports list the vaccine as “unknown”, thus these reports are excluded in some parts of our data analysis. Also, among the reported events or symptoms, some of them are adverse, while some are not considered to be adverse, such as drug ineffective, inappropriate schedule of drug administration, unevaluable event, wrong drug administration, full blood count, full blood count normal, etc. In the parts of our analysis on the relationship between the vaccines and the *adverse* events or symptoms, we exclude those vectors in Eq. (1) if *Z* is a non-adverse event or symptom.

#### Top 100 Adverse Symptoms

Due to the large size of the dataset being considered in this research and due to our limited computing power, parts of our analysis here focus on the cross-board patterns of how those most frequently occurred *adverse* symptoms or events are related to the vaccines, because it would take several weeks to complete just one explorative data visualization plot for all 7368 symptoms due to its large dimension. Specifically, excluding those non-adverse events or symptoms aforementioned, the top 100 *adverse* symptoms or events with highest overall occurrence frequencies in the processed VAERS dataset (1) are identified and listed in Table 1, where *Z*_1_ is the adverse symptom with the highest occurrence frequency in the dataset, *Z*_2_ is the adverse symptom with the 2nd highest occurrence frequency in the dataset, and so forth; and *FQ*_*i*_ is the total occurence frequency for symptom *Z*_*i*_. Hereafter in this article, these are referred as the *top 100 adverse symptoms*. We note that among top 107 events or symptoms with highest overall occurrence frequencies, seven are non-adverse, thus Table 1 does not include these 7 non-adverse events.

**Table 1:** List of Top 100 Adverse Symptoms

*i*	*Z*_*i*_	*F* *Q*_*i*_	*i*	*Z*_*i*_	*F* *Q*_*i*_	*i*	*Z*_*i*_	*F* *Q*_*i*_
1	Pyrexia	138934	35	Injection site mass	15186	69	Blister	6100
2	Injection site erythema	82620	36	Cough	14802	70	Chest pain	6083
3	Rash	56780	37	Fatigue	14007	71	Loss of consciousness	6025
4	Injection site swelling	48210	38	Cellulitis	13798	72	Rash macular	5905
5	Erythema	48062	39	Malaise	13365	73	Insomnia	5865
6	Injection site pain	47536	40	Injection site reaction	12359	74	Musculoskeletal stiffness	5765
7	Pain	41738	41	Tremor	12234	75	Pharyngitis	5698
8	Urticaria	37259	42	Syncope	12169	76	Laboratory test abnormal	5631
9	Vomiting	37137	43	Somnolence	11731	77	Decreased appetite	5570
10	Injection site oedema	35952	44	Feeling hot	11700	78	Herpes zoster	5523
11	Pruritus	34937	45	Oedema	11584	79	Back pain	5436
12	Headache	31439	46	Paraesthesia	11329	80	Face oedema	5370
13	Injection site warmth	30571	47	Rash maculo-papular	11287	81	Rash generalised	5323
14	Injection site hypersensitive	27569	48	Skin warm	10211	82	Otitis media	5314
15	Dizziness	24429	49	Hypotonia	9627	83	Apnoea	5274
16	Crying	24345	50	Rash erythematous	9008	84	Neck pain	5254
17	Agitation	24058	51	Body temperature increase	8889	85	Gait disturbance	5105
18	Convulsion	23891	52	Hyperhidrosis	8345	86	Gaze palsy	5092
19	Nausea	23694	53	Lymphadenopathy	8329	87	Condition aggravated	4884
20	Oedema peripheral	23154	54	Hypoaesthesia	8326	88	Immed. post-injection reaction	4850
21	Diarrhoea	19343	55	Tenderness	8261	89	White bloodcell count increase	4775
22	Injection site induration	18762	56	Anorexia	8151	90	Wheezing	4752
23	Dyspnoea	18074	57	Hypersensitivity	8148	91	Rash vesicular	4740
24	Screaming	17991	58	Injection site pruitus	7500	92	Muscle twitching	4684
25	Myalgia	17983	59	Hypokinesia	7494	93	Rhinorrhoea	4648
26	Chills	17244	60	Dermatitis bullous	7310	94	Muscular weakness	4515
27	Pain in extremity	16667	61	Febrile convulsion	7226	95	Rhinitis	4509
28	Swelling	16439	62	Abdominal pain	7081	96	Dyskinesia	4135
29	Infection	16313	63	Injection site rash	7076	97	Skin ulcer	4016
30	Vasodilatation	16254	64	Lethargy	7025	98	Hypertonia	4003
31	Pallor	16001	65	Cyanosis	6953	99	Rash pruritic	3988
32	Asthenia	15661	66	Stupor	6798	100	Skin discolouration	3835
33	Irritability	15476	67	Rash papular	6415			
34	Arthralgia	15208	68	Viral infection	6295			

## Data visualization and statistical analysis

In addition to the large size issue, the analysis of VAERS data deals with nominal variables such as vaccines and events or symptoms; in particular, the symptom is a nominal variable of very large dimension. Here, we use data visualization methods in our studies.

**Fig. 1:**
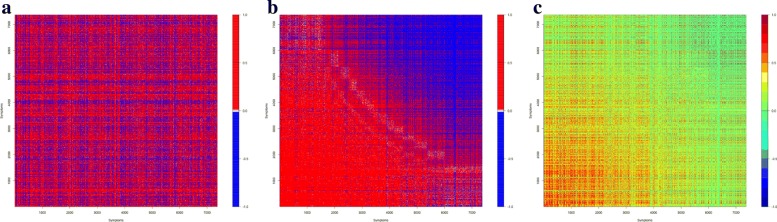
Correlation matrix of all reported events

For an initial data visualization, we consider all different *n*=7368 events or symptoms reported in processed VAERS dataset (1) and arrange them according to the alphabetical order: *E*_1_,*E*_2_,⋯,*E*_*n*_. We denote all reported 72 vaccines according to the following order:

2 $$ V_{1}, V_{2}, \cdots, V_{72}  $$

where *V*_1_,⋯,*V*_24_ are alphabetically ordered 24 bacteria vaccines, *V*_25_,⋯,*V*_62_ are alphabetically ordered 38 virus vaccines, *V*_63_,⋯,*V*_71_ are alphabetically ordered 9 bacteria/virus combined vaccines, and *V*_72_ represents the vaccine listed as *unknown*. For each vaccine *V*_*k*_, we obtain the frequency vector ***X***_*k*_=(*X*_*k*1_,*X*_*k*2_,⋯,*X*_*kn*_), where *n*=7,368 and *X*_*ki*_ is the total number of times that event *E*_*i*_ was reported for vaccine *V*_*k*_. Based on these 72 vectors ***X***_*k*_, we compute the rotated 7368×7368 matrix of sample correlation coefficients:

3 $$ {{} \begin{aligned} \hat{\rho}_{ij} &= \frac{\sum^{72}_{k=1}\left(X_{ki} - \bar{X}_{i}\right)\left(X_{kj} - \bar{X}_{j}\right)} {\sqrt{\sum^{72}_{k=1}\left(X_{ki} - \bar{X}_{i}\right)^{2}}\, \sqrt{\sum^{72}_{k=1}\left(X_{kj} - \bar{X}_{j}\right)^{2}}},\\& \qquad i, j = 1, 2, \cdots, 7368 \end{aligned}}  $$

where $\bar {X}_{i}$ is the sample mean of *X*_1,*i*_,⋯,*X*_72,*i*_, and $\hat {\rho }_{ij}$ is the sample correlation coefficient of symptoms *E*_*i*_ and *E*_*j*_. This matrix is displayed in Fig. 1a, where red dots represent for those $\hat {\rho }_{ij} > 0.01$, white dots for $|\hat {\rho }_{ij}|\le 0.01$, and blue dots for $\hat {\rho }_{ij} < -0.01$. Throughout this article, all matrices are displayed as the rotated version of the conventional matrix, i.e., with the bottom row of the conventional matrix as the top row here. Obviously, Fig. 1a shows no informative patterns about the dataset.

Next, we denote all reported symptoms or events in VAERS data (1) by: $\mathbb {E}_{1}, \mathbb {E}_{2}, \cdots, \mathbb {E}_{n}$, where $\mathbb {E}_{1}$ is the symptom or event with the highest occurrence frequency in the dataset, $\mathbb {E}_{2}$ is the symptom or event with the 2nd highest occurrence frequency in the dataset, and so forth. For each vaccine *V*_*k*_ in (2), we obtain the frequency vector ***Y***_*k*_=(*Y*_*k*1_,*Y*_*k*2_,⋯,*Y*_*kn*_), where *Y*_*ki*_ is the total number of times that event $\mathbb {E}_{i}$ was reported for vaccine *V*_*k*_. Based on such 72 vectors ***Y***_*k*_, we compute the rotated matrix of sample correlation coefficients $\hat {\rho }_{ij}^{Y}$ using the formula in (3) for *Y*_*ki*_’s, where $\hat {\rho }_{ij}^{Y}$ is the sample correlation coefficient of symptoms $\mathbb {E}_{i}$ and $\mathbb {E}_{j}$. This matrix is displayed in Fig. 1b, where the colored dots have the same meaning for $\hat {\rho }^{Y}_{ij}$ as for those in Fig. 1a. In addition, Fig. 1c displays the matrix of Fig. 1b with 20 different colors to illustrate the values of the sample correlation coefficients $\hat {\rho }^{Y}_{ij}$, where green color corresponds to values of $\hat {\rho }^{Y}_{ij}$ around 0, color from green to red corresponds to $\hat {\rho }^{Y}_{ij} > 0$, and color from green to blue corresponds to $\hat {\rho }^{Y}_{ij} < 0$. Interestingly, such a method of data visualization clearly indicates cross-board patterns.

**Fig. 2:**
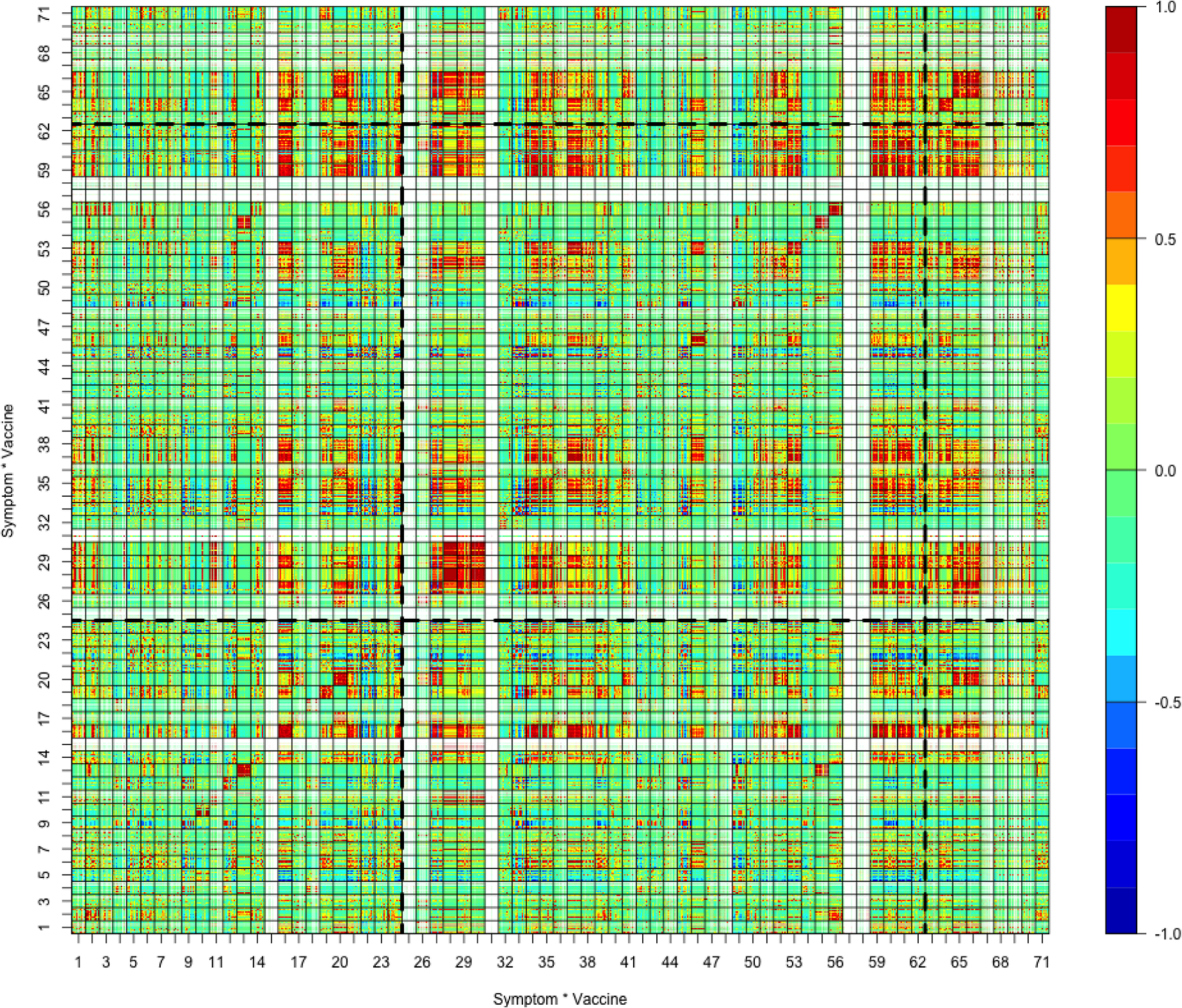
Correlation matrix of top 100 adverse symptoms under vaccines

For the study of the cross-board patterns on the relationship between the vaccines and the *adverse* events or symptoms, we consider the top 100 adverse symptoms *Z*_1_,⋯,*Z*_100_ listed in Table 1, and consider the vaccines *V*_1_,⋯,*V*_71_ listed in (2); that is in our analysis hereafter we exclude those vectors in processed VAERS dataset (1) that list the vaccine as “unknown”. For each year, we obtain frequency vector ***F***_*k*_=(*F*_*k*,1,1_,⋯,*F*_*k*,1,100_,*F*_*k*,2,1_,⋯,*F*_*k*,2,100_,⋯*F*_*k*,71,100_), where *k*=1,⋯,24 represent 24 years between 1990–2013; and *F*_*kij*_ is the total number of times that symptom *Z*_*j*_ was reported for vaccine *V*_*i*_ during year *k*. Based on these 24 vectors ***F***_*k*_, we compute the rotated 7100×7100 matrix of sample correlation coefficients $\hat {\rho }_{ij,lq}$ using the formula in (3) for *F*_*kij*_’s, where $\hat {\rho }_{ij,lq}$ is the sample correlation coefficient of symptom *Z*_*j*_ under vaccine *V*_*i*_ and symptom *Z*_*q*_ under vaccine *V*_*l*_, thus $\hat {\rho }_{ij,iq}$ is the sample correlation coefficient of symptoms *Z*_*j*_ and *Z*_*q*_ under vaccine *V*_*i*_. This matrix is displayed in Fig. 2, where the colored dots have the same meaning for $\hat {\rho }_{ij,lq}$ as for those in Fig. 1c.

As indicated by solid lines, the matrix in Fig. 2 consists of 71^2^=5041 block matrices ***M***_*ij*_, each of which is of dimension 100×100 and is the matrix of sample correlation coefficients of top 100 adverse symptoms under vaccines *V*_*i*_ and *V*_*j*_. For *i*≠*j*, the block matrices ***M***_*ij*_ and ***M***_*ji*_ satisfy $\boldsymbol {M}_{ij}^{\top }=\boldsymbol {M}_{ji}$, while ***M***_*ii*_ is the matrix of sample correlation coefficients of top 100 adverse symptoms under vaccine *V*_*i*_ and is a block matrix located on the diagonal line of the matrix in the direction from bottom left to top right.

**Fig. 3:**
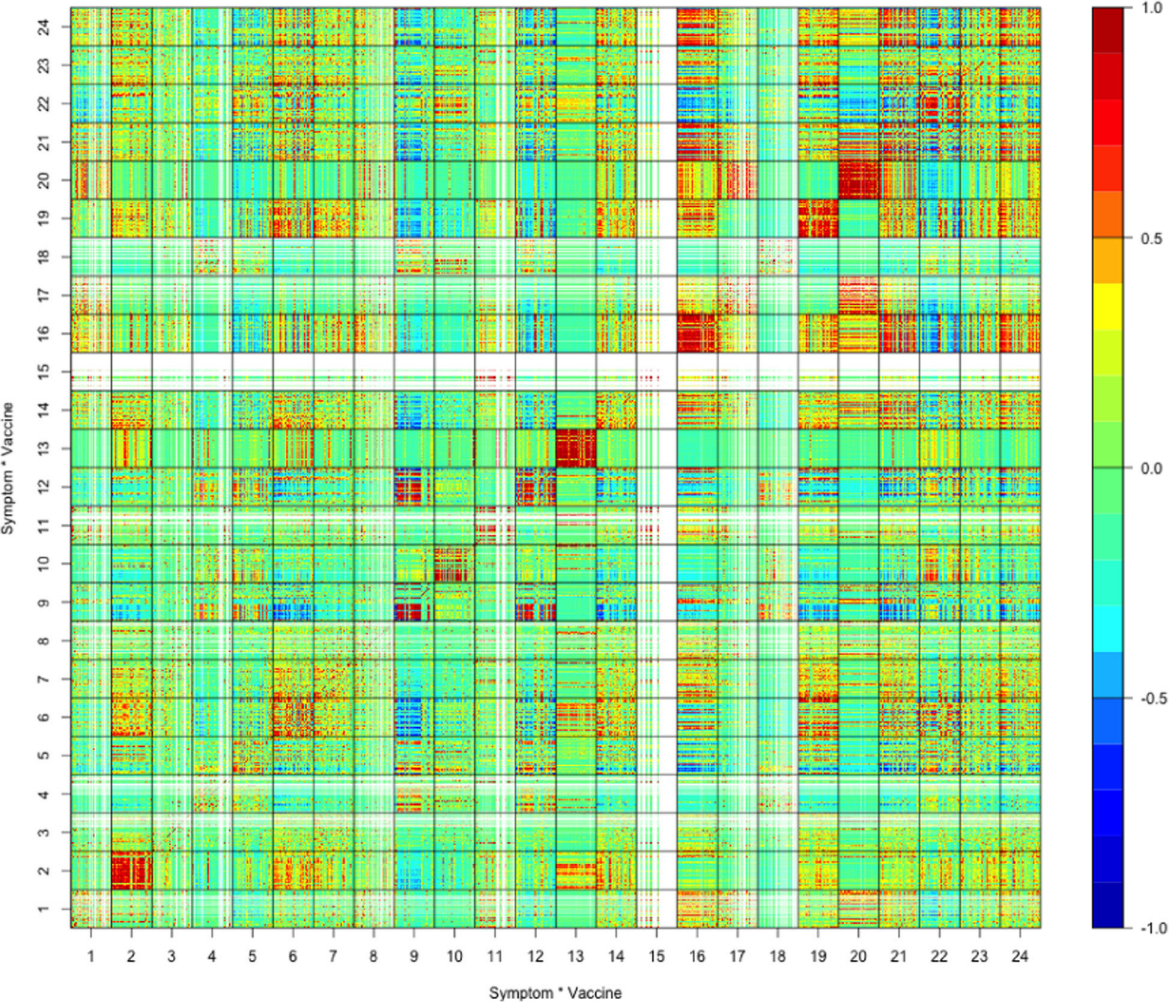
Correlation matrix under bacteria vaccines

Due to the order of vaccines *V*_*i*_’s in (2), the bold dashed lines separate the matrix of Fig. 2 into 9 big block matrices, among which the square block matrix in the bottom left, displayed separately in Fig. 3, is the matrix of sample correlation coefficients of top 100 adverse symptoms under all 24 different bacteria vaccines; and the square block matrix in the middle, displayed separately in Fig. 5, is the the matrix of sample correlation coefficients of top 100 adverse symptoms under all 38 different virus vaccines.

**Fig. 4:**
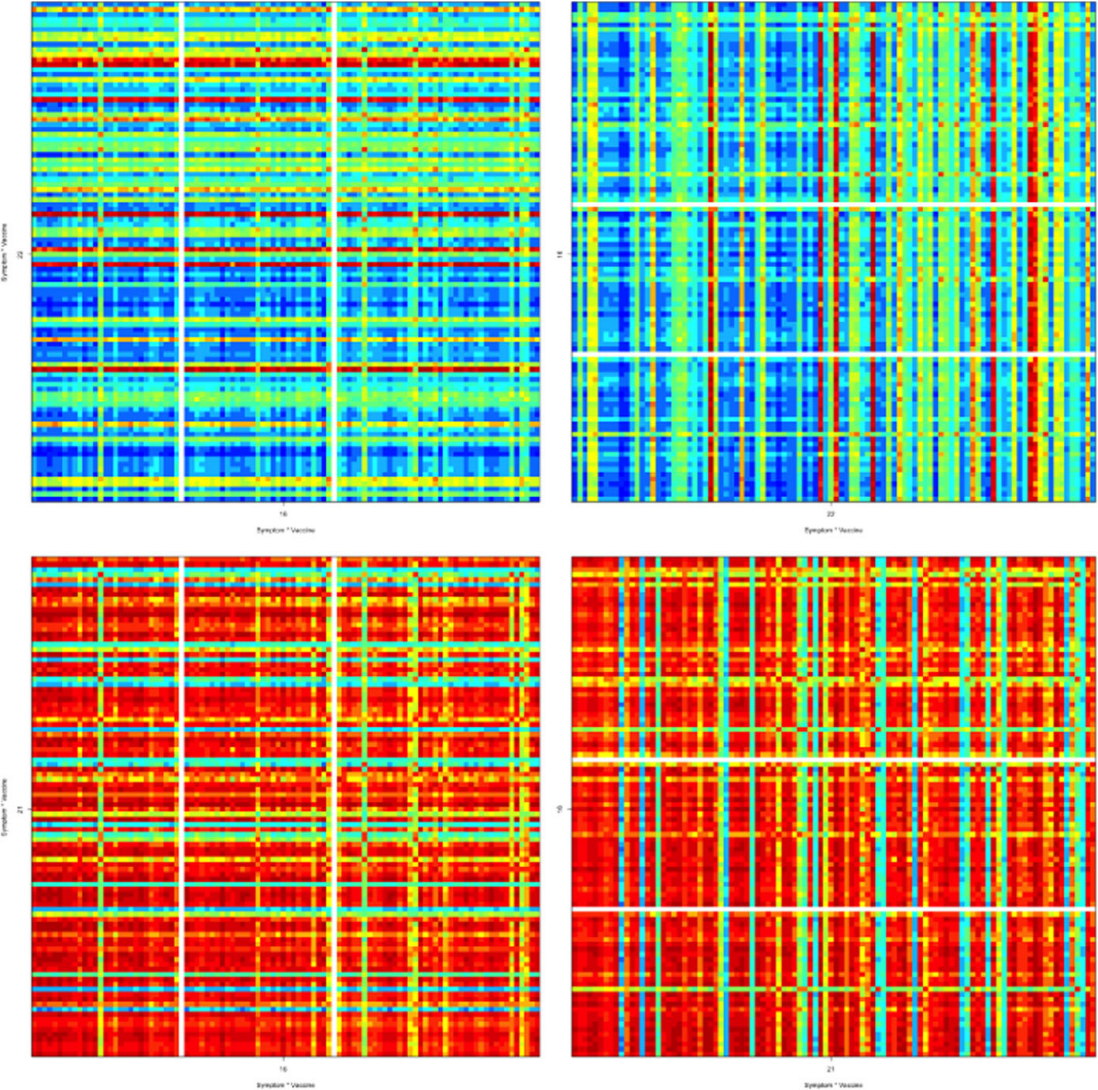
Four Block Matrices of Fig. 3

In Fig. 4, the top are block matrices ***M***_16,22_ and ***M***_22,16_ in Fig. 3, and the bottom are block matrices ***M***_16,21_ and ***M***_21,16_ in Fig. 3. Due to better picture resolution reason, these block matrices clearly show that equation $\boldsymbol {M}_{ij}^{\top }=\boldsymbol {M}_{ji}$ holds. The two block matrices on the top of Fig. 4 are among those mostly green-blue colored block matrices in Fig. 3, while the two block matrices on the bottom are the very few non-diagonal block matrices in Fig. 3 that are mostly red colored.

**Fig. 5:**
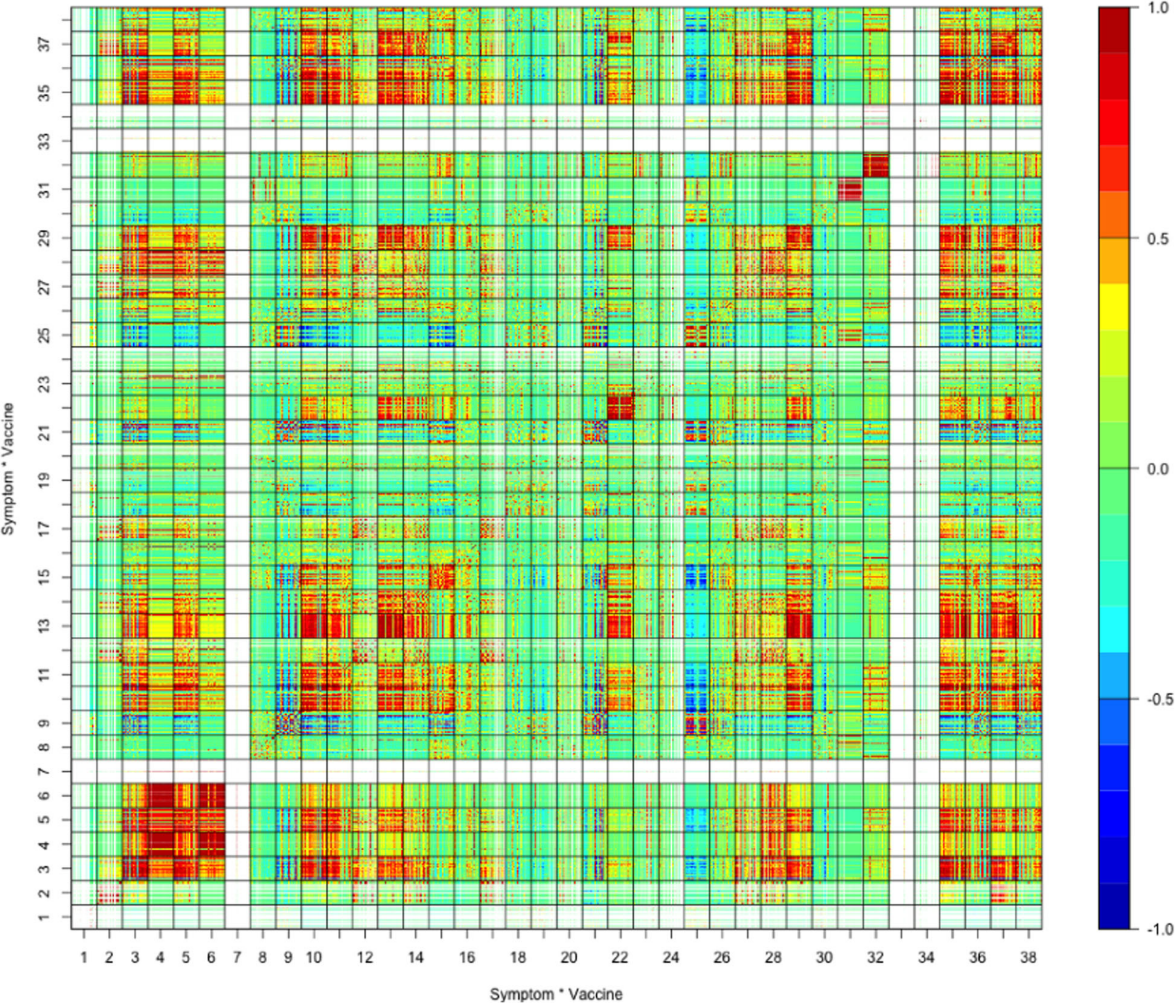
Correlation matrix under virus vaccines

**Fig. 6:**
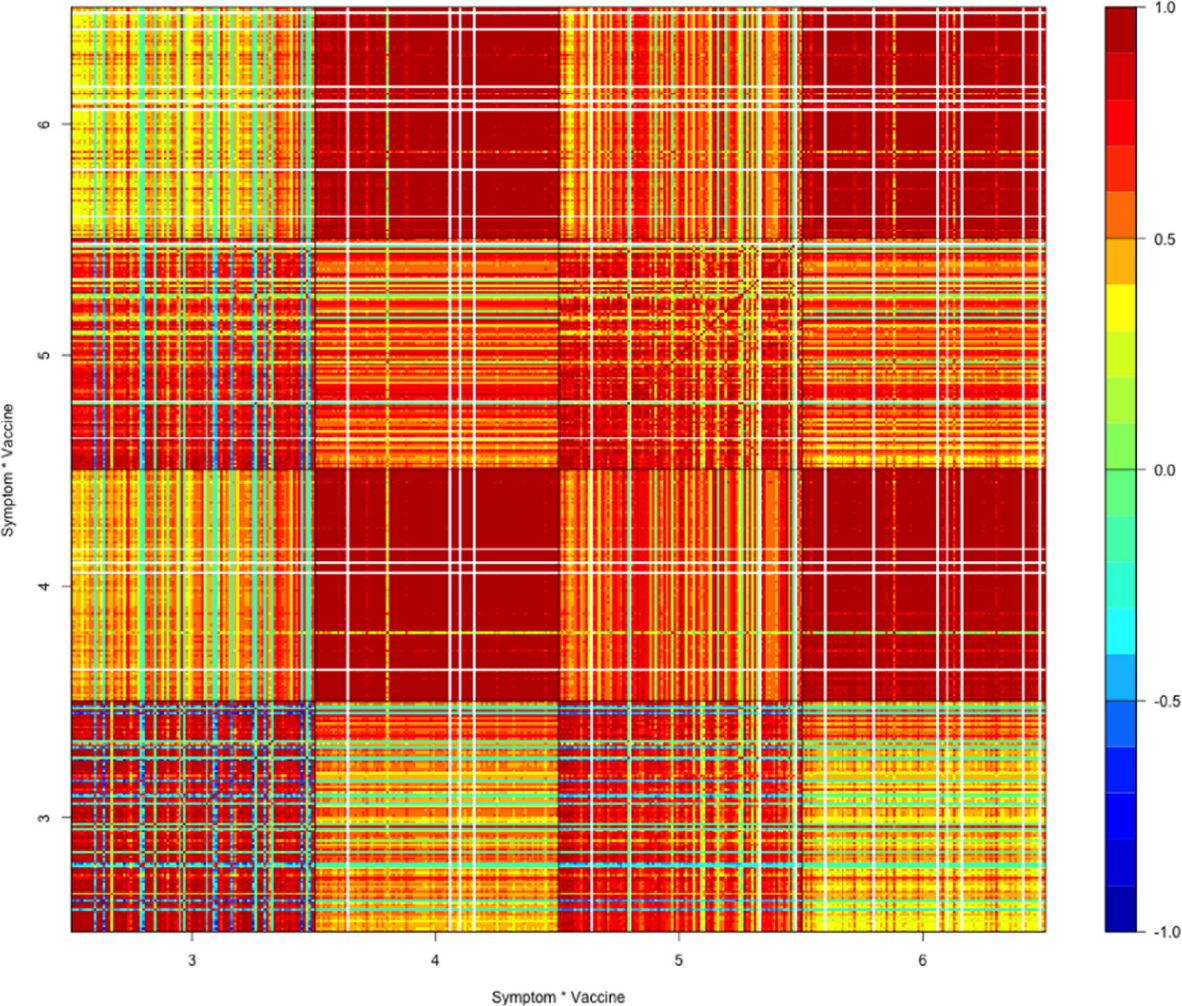
Block matrices of Fig. 5 under Flu vaccines

Figure 6 contains the block matrices ***M***_*ij*_ of Fig. 5 for *i*,*j*=3,4,5,6, which are the correlation matrices for the top 100 adverse symptoms under 4 different flu vaccines: FLU, FLU(H1N1), FLUN and FLUN(H1N1).

**Fig. 7:**
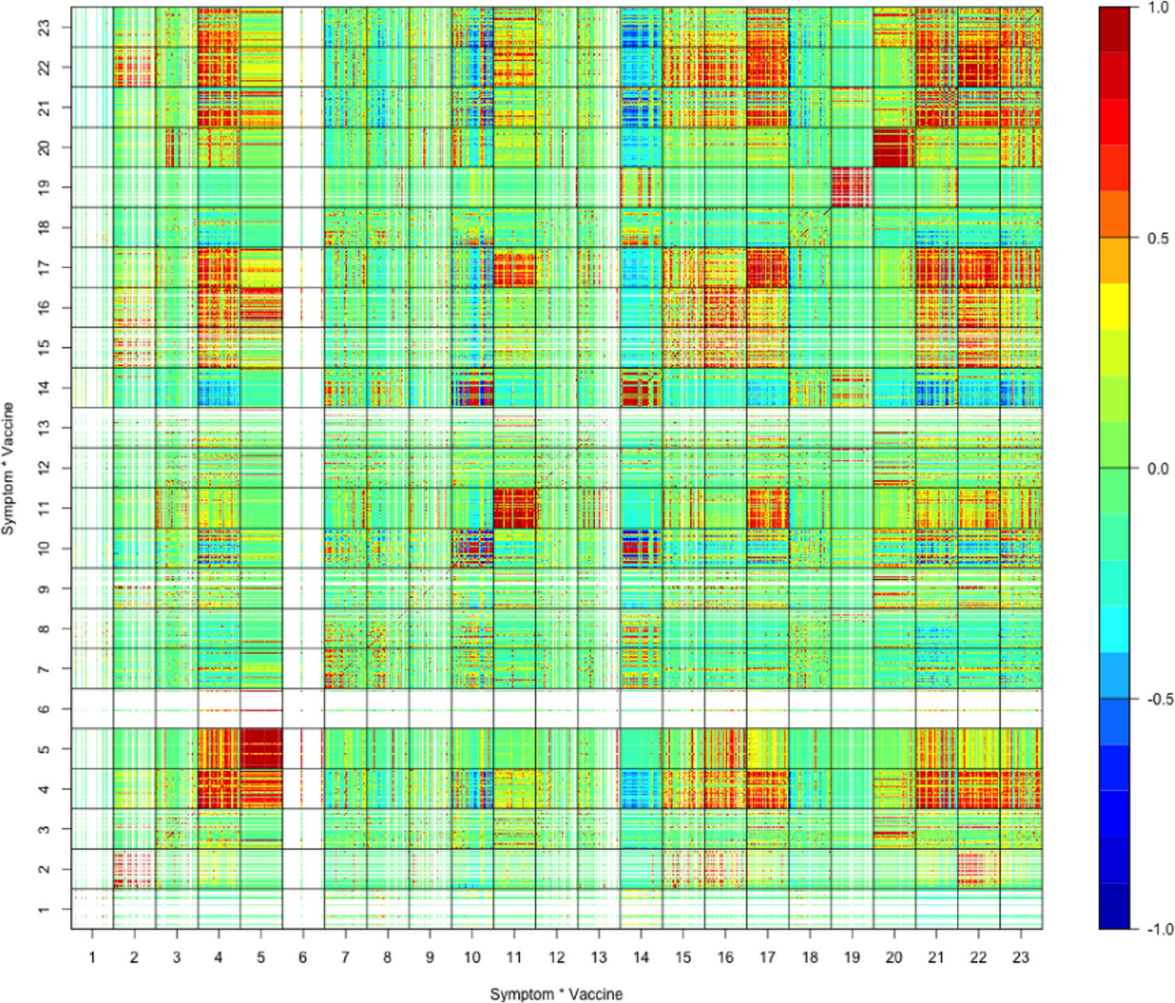
Correlation matrix under live vaccines

**Fig. 8:**
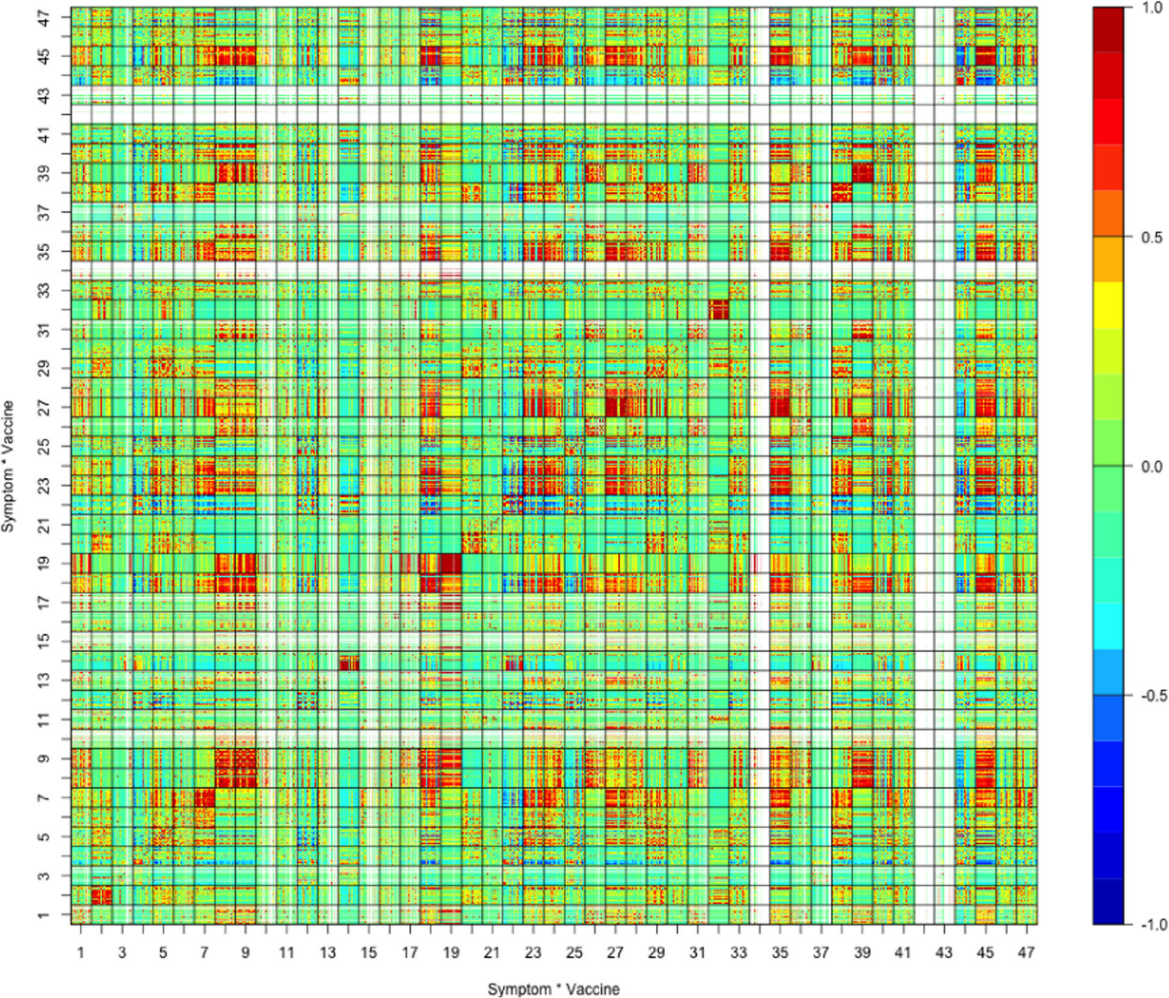
Correlation matrix under inactivated vaccines

For the study of the relations between vaccine-adverse events and attributes of vaccines, such as live attenuated vaccine vs. killed inactivated vaccine, Fig. 7 displays the matrix of sample correlation coefficients of top 100 adverse symptoms under all 23 different live vaccines in processed VAERS dataset (1), while Fig. 8 displays the matrix of sample correlation coefficients of top 100 adverse symptoms under all 47 different inactive vaccines.

## Results

Figure 1b shows that over all reported vaccines, those reported events or symptoms (adverse or non-adverse) with overall high occurrence frequencies are positively correlated, while those with low occurrence frequencies are negatively correlated. In comparison, the blue area of Fig. 1b mostly shows green color in Fig. 1c, which, by color design, indicates that the low-occurrence events or symptoms are mostly uncorrelated.

Figure 3 shows that the top 100 adverse symptoms listed in Table 1 are mostly uncorrelated or negatively correlated under different bacteria vaccines. Also, the big rectangular block matrix in the bottom middle of Fig. 2 outlined by the bold dashed lines are mostly green-blue colored, except the row block #16 (bacteria vaccine MNQ), which indicates that the top 100 adverse symptoms under bacteria vaccines are mostly uncorrelated or negatively correlated with the top 100 adverse symptoms under virus vaccines.

Figures 5 and 6 show that the top 100 adverse symptoms are in many cases positively correlated under different virus vaccines, especially under flu vaccines. In particular, Fig. 6 shows that the top 100 adverse symptoms are strongly positively correlated under vaccines FLU and FLUN, and they are even more strongly positively correlated under vaccines FLU(H1N1) and FLUN(H1N1).

Figures 7 and 8 show that under different live or inactive vaccines, the top 100 adverse symptoms are in some cases positively correlated and in some cases negatively correlated, because in both figures many mostly red or mostly blue non-diagonal block matrices are scattered all over the places.

### Summary

The results of our analysis indicate: (a) Over all reported vaccines, those events or symptoms (adverse or non-adverse) with overall high occurrence frequencies are positively correlated, while those with low occurrence frequencies are uncorrelated; (b) Those most frequently occurred *adverse* symptoms or events are mostly uncorrelated or negatively correlated under different bacteria vaccines, but they are in many cases positively correlated under different virus vaccines, especially under flu vaccines; (c) Under different live or inactive vaccines, those most frequently occurred *adverse* symptoms or events are in some cases positively correlated and in some cases negatively correlated.

## Discussion

The FDA VAERS database provides useful information for the analysis of the relations between the vaccines and the adverse events or symptoms. However, the dataset is huge, includes reports with multiple listing of vaccines and adverse symptoms in a single report, and contains reports with errors or incomplete information. Using our proposed neighboring method for processing the raw VAERS data coupled with novel and proper utilization of data visualization techniques (arbitrary use of data visualization obviously does not work, eg., Fig. 1a), here we conclusively reveal some interesting cross-board patterns for those most frequently occurred adverse symptoms or events under bacteria vaccines vs virus vaccines as well as under live vaccines vs inactive vaccines. Our findings here suggest some insights and the direction of further studies on certain vaccines and related adverse symptoms.

For instance, our finding of the low-occurrence events or symptoms’ being mostly uncorrelated may be interpreted as that the rarely occurred events or symptoms are mainly vaccine-specific, they generally are not associated among one another, thus are not onset as a cluster. Also, although Fig. 3 shows that the top 100 adverse symptoms are mostly uncorrelated or negatively correlated under different bacteria vaccines, the block matrices ***M***_16,21_ and ***M***_21,16_ in Fig. 4 show that they are, as an isolated case, very much positively correlated under bacteria vaccines MNQ (#16, Meningococcal Vaccine Menactra) and PPV (#21, Pneumococcal Polysaccharide Vaccine). Moreover, although as shown by the big rectangular block matrix in the bottom middle of Fig. 2, the top 100 adverse symptoms under bacteria vaccines are mostly uncorrelated or negatively correlated with the top 100 adverse symptoms under virus vaccines, the row block #16 (bacteria vaccine MNQ) of this big rectangular block matrix indicates that the top 100 adverse symptoms under bacteria vaccine MNQ are positively correlated with those under many virus vaccines.

Interestingly, as shown in Fig. 6, the top 100 adverse symptoms are strongly positively correlated under FLU (inactivated flu vaccine, virus vaccine) and FLUN (live flu vaccine), but not as strong as those under FLU(H1N1) and FLUN(H1N1). Such difference is likely due to the fact that FLU and FLUN are typically prepared using three flu viruses: an influenza A (H1N1) virus, an influenza A (H3N2) virus, and an influenza B virus. However, FLU(H1N1) and FLUN(H1N1) are prepared with only one influenza A (H1N1) virus.

In addition to the differences between live vs inactivated vaccines and between bacterial and viral vaccine types which have been considered in this article, other factors such as whole organism vs subunit vaccines, etc., may also affect the outcome of adverse events or symptoms. Further investigation and data analysis on VAERS data are needed.

## Conclusions

In this article, we identify certain cross-board patterns of the relationship between the vaccines and the reported events or symptoms via the combined approaches based on our proposed neighboring method and novel utilization of data visualization techniques. This is useful for better understanding the VAERS data, and shows that the data visualization method, if used properly, can serve as a helpful tool for big data analysis problems involving large dimension nominal variables. Moreover, what is discovered in this article provides a needed starting point for the development of statistical models and procedures to further analyze the VAERS data. In fact, a statistical methodology paper (Ren and Sun: An empirical likelihood based NROC classification procedure, in preparation) based on the results here is forthcoming. The ultimate goal is using reliable statistical analysis to help detect and monitor the adverse events or symptoms after vaccination in the years to come.

## Abbreviations

MNQ: Meningococcal vaccine menactra
PPV: Pneumococcal Polysaccharide vaccine
VAERS: Vaccine adverse event reporting system
